# Nearshore neonate dispersal of Atlantic leatherback turtles (*Dermochelys coriacea*) from a non-recovering subpopulation

**DOI:** 10.1038/s41598-020-75769-0

**Published:** 2020-10-30

**Authors:** Aimee L. Hoover, George L. Shillinger, Sean A. Williamson, Richard D. Reina, Helen Bailey

**Affiliations:** 1 Chesapeake Biological Laboratory, University of Maryland Center for Environmental Science, Solomons, MD USA; 2 Upwell, Monterey, CA USA; 3 School of Biological Sciences, Monash University, Clayton, VIC Australia

**Keywords:** Ecology, Zoology, Ecology, Ocean sciences

## Abstract

The cryptic ‘lost years’ of sea turtles challenge conservation efforts due to unknown movements and habitat utilisation of young life stages. Behavioural information strengthens dispersal and habitat utilisation models estimating unidentified movements. In this study, leatherback hatchlings were actively tracked with miniature acoustic tags off the east coast of Costa Rica for 83.15 min (± 9.12 SD) to determine their movements and swimming behaviour. Drifters were deployed throughout the tracking process to obtain surface current data. Hatchling (n = 42) over-ground and in-water swimming speed and bearing were calculated. Mean over-ground distance travelled was 2.03 km (± 0.71 km SD) with an over-ground average swim speed of 0.41 m/s (± 0.15 m/s SD). Mean bearing was 108.08° (± 20.19° SD) compared to the 137.56° (± 44.00° SD) bearing of nearshore ocean currents during tracking. Hatchling mean in-water swimming speed was 0.25 m/s (± 0.09 m/s SD). The lower in-water speed suggests hatchlings were advected by the currents, with overall movement strongly influenced by the current direction. This information can be assimilated into broader spatiotemporal distribution models to interpret the influence of directional swimming on ecosystem utilisation and help to achieve informed management decisions across all life stages of the population.

## Introduction

Highly migratory marine species have complex conservation needs and pose management challenges^1^, which includes sea turtles that undertake long distance oceanic migrations^2^. Management is further complicated by the ‘lost years’ life stage of sea turtles, the time after which hatchlings depart from the nesting beaches, develop in unknown habitats, and eventually return at maturation to breed. Adult leatherback turtles (*Dermochelys coriacea*) have the widest reptilian distribution, but have largely unknown movements and nursery habitats during young life stages^3^.

Many leatherback turtle populations have experienced dramatic declines in recent decades^4,5^, with substantial losses are often attributed to fisheries bycatch, pollution, climate change, nesting beach degradation, and poaching of eggs and adults^6^. To prevent extirpation of declining populations and ensure the future of stable or recovering populations, modelling efforts on the dispersal and habitat utilisation throughout life stages aim to increase understanding of population distributions. These biophysical models are based on the historical premise of denatant dispersal, where the young of species passively drift with winds and currents from hatching to nursery areas and until recently, did not include behavioural information, such as orientation and speed of hatchling turtles^7–9^. However, both swimming and currents influence the ultimate dispersal outcome of hatchlings^10–13^. Research has recently taken place to begin understanding the active movements of sea turtles during the ‘lost years’ period e.g.^10,14,15^, although this has generally been for hard-shelled sea turtles, and there is little empirical data for young leatherbacks, with juvenile speed estimates derived from tagged adults^16^.

Dispersal during the hatchling frenzy period, a period of continuous swimming, must be efficient and directed to prevent predation and entrainment in coastal waters^17–19^. These dispersal outcomes can be influenced by even small active movements within moving water masses in strong currents, and these outcomes influence travel to developmental habitats and population dynamics^11,14,20^. Knowledge gaps on this role of active movement still persist, especially for leatherback turtles during their most vulnerable hatchling stage^2^. The inherent small size of hatchling turtles increases the difficulty of obtaining long-term observations because technologies commonly deployed in movement studies, such as satellite tags, are still too large for these small individuals to carry. Therefore, direct field observations and short-term experiments remain the best method for attaining these data.

The distinct Northwest Atlantic leatherback turtle population is classified as endangered on the U.S. Endangered Species Act and Vulnerable on the IUCN Red List. The Costa Rican rookery of this population has not experienced the recovery documented in other nesting locations^5^, and disproportionately high fisheries bycatch in the Gulf of Mexico may be one source of this downward trend^21^. Acoustic telemetry has been successfully employed to track other hatchling sea turtle species^8,9,22,23^, with no known published tracks from attempts to track leatherback turtles^24^. Here, we examine nearshore dispersal of hatchlings from the leatherback nesting population at Pacuare Nature Reserve, Costa Rica using active acoustic tracking to determine their movement from the natal beach. Field experiments were undertaken to obtain *in-situ* observations of individual Atlantic leatherback hatchling movements to improve our knowledge of their behaviour and dispersal. The specific objectives were to (1) test whether the line-float-transmitter attachment protocol and acoustic tracking methods could be used for tracking hatchling leatherbacks at sea, (2) acoustically track individual hatchling leatherbacks for insight into initial movements (speed and bearing) after natal beach departure, a novel approach for leatherbacks, and (3) examine local, fine-scale oceanic conditions encountered by the hatchling leatherbacks and the influence of these conditions on their initial at-sea movements.

## Results

### Hatchling movements

**Figure 1:**
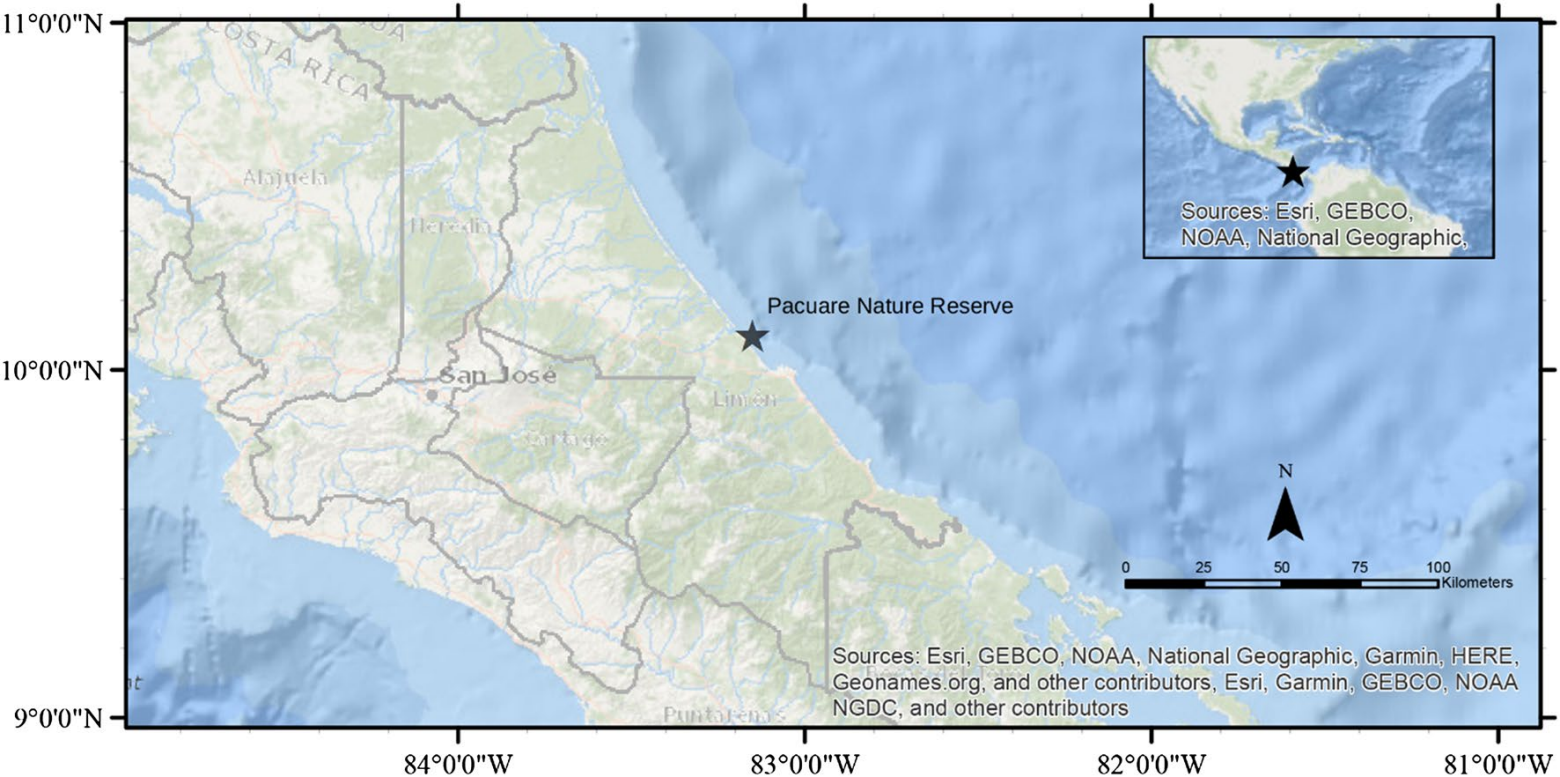
Map of hatchling acoustic tracking location at Pacuare Nature Reserve on the Atlantic Coast. Pacuare Nature Reserve is part of a continuous stretch of nesting beaches for Northwest Atlantic leatherbacks. The map was generated using ArcGIS Desktop, ESRI, v.10.1 (https://desktop.arcgis.com).

Forty-three hatchlings were obtained from hatchery (n = 22), incubator-reared (n = 15), and relocated (n = 6) nests at the Pacuare Nature Reserve, Limón Province, Costa Rica (Fig. 1). The mean weight of the leatherback turtles tagged was 42.5 g (± 3.5 g SD), with a mean standard carapace width of 41.8 mm (± 1.6 mm SD), mean standard carapace length of 60.4 mm (± 3.6 mm SD), and 17.8 mm (± 0.6 mm SD) head width. One track of the 43 hatchlings was interrupted within the starting 30-min window set for inclusion in the analysis, an approximately 98% success rate with 42 successful tracks.

Predation was of high concern given previous hatchling studies^9,19,22^. However, only one hatchling was successfully predated by a tarpon at 85 min during the tracking. The line-float-transmitter attachment on this predated turtle was rejected by the tarpon and recovered. In an unsuccessful predation attempt, the hatchling excluded from analysis was attacked by a frigate bird prior to reaching the 30-min minimum track length. The turtle was recovered with a small net, the line-float-transmitter attachment (Fig. S1) was removed, and it was released without apparent injuries. After each hatchling was tracked, the drifters were recovered (Fig. S2). The mean bathymetry during the tracking was 10.39 m (± 4.36 m SD) (Fig. S3), and the mean water temperature recorded from the Microstar drifter was 29.7 °C (± 0.8 °C SD).

**Figure 2:**
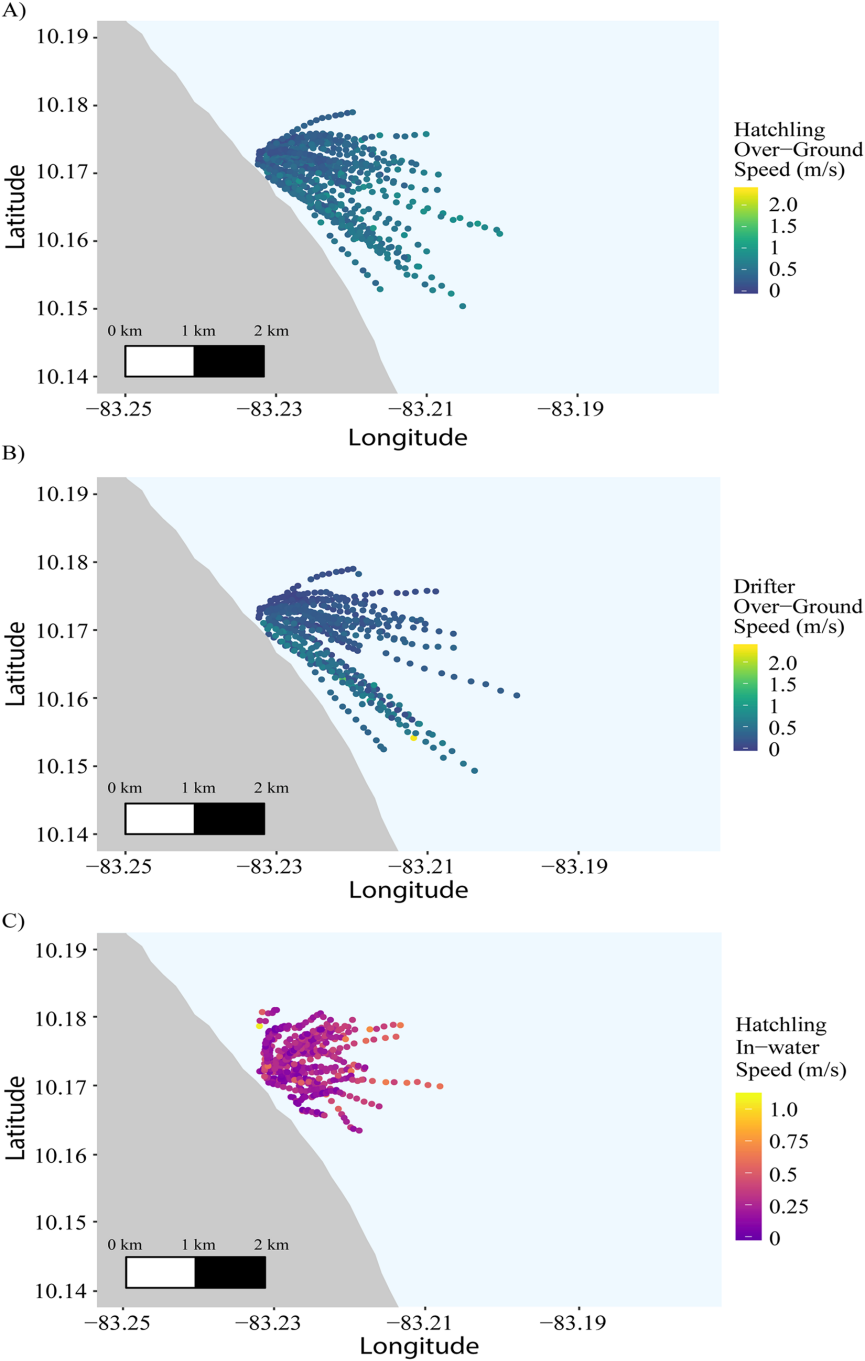
Trajectories of (**A**) hatchlings and (**B**) surface drifters by over-ground speed (m/s) and (**C**) track-corrected hatchlings by in-water speed (m/s). Hatchlings and drifters were released outside the surf zone near Pacuare Nature Reserve, Costa Rica in August and September 2016.

Over the entire tracking period, hatchlings were actively tracked for a mean of 83.15 min (± 9.12 min SD) with a mean detection rate of 2.93 detections/min (± 0.82 detections/min SD). Distances travelled were 0.65–3.70 km for hatchlings and 0.30–3.91 km for the drifters (Table 1). Mean over-ground swimming speed of hatchlings was 0.41 m/s (± 0.15 m/s SD) (Fig. 2A; Fig. S4). This is equivalent to approximately 6.79 body lengths per second. The mean hatchling bearing was 108.08° (± 20.19° SD). An average hatchling compass heading of 44° (± 23°; median = 40°) was recorded, a north-east trajectory.

**Table 1:** Distances travelled (km) and over-ground speed (m/s) of leatherback hatchlings and drifters deployed from Pacuare Nature Reserve, Costa Rica.

	Min dist (km)	Max dist (km)	Mean dist (km)	Dist SD (km)	Median dist (km)	Mean time (s)	Mean speed (m/s)	Speed SD (m/s)
Leatherback hatchling	0.65	3.70	2.03	0.71	2.00	4988.91	0.41	0.15
Combined drifters	0.30	3.91	1.43	0.88	1.32	5173.55	0.29	0.19
Microstar drifter	0.11	2.61	1.18	0.72	1.17	4460.00	0.28	0.18
Mobile phone drifter	0.40	2.02	0.91	0.61	0.62	2658.00	0.36	0.26

Mean times are provided in seconds, along with the mean, standard deviation, and median distances travelled. Dist stands for distance, Min for minimum, Max for maximum, and SD for standard deviation.

Time was a significant explanatory variable for the transformed over-ground speed of hatchlings (p < 0.0001; Table S1 and Fig. S5A), while bathymetry, tidal state, and hatchling treatment were not statistically significant (α = 0.05). Time intervals 8 and 10–17 (representing the period immediately before and all those proceeding the halfway point of tracking) were weakly associated with significantly higher over-ground speed (R^2^_:_ marginal = 0.067, conditional = 0.68; Table S2).

Both time and tidal state were significant fixed effects for bearing of hatchlings (p < 0.0001; Table S1), with bathymetry and hatchling treatment not statistically significant. A high tidal state resulted in significantly higher hatchling bearing (p = 0.0006; Table S2). Later time periods during the study, after the halfway point of each track (time intervals 10–17), resulted in significantly higher hatchling bearing (R^2^_:_ marginal = 0.13, conditional = 0.48; Table S2 and Fig. S5B).

### Effects of surface currents on swimming speed

A Pacific Gyre Microstar drifter was deployed at the beginning of tracking 31 turtles, and a secondary drifter was deployed during a subset of these tracks (n = 10) at approximately 45 min into tracking (Fig. S6). The mean Microstar drifter bearing was 134.75° (± 43.77° SD) with an average speed of 0.28 m/s (± 0.18 m/s SD), and the mean secondary drifter bearing of 157.07° (± 18.57° SD) with a mean speed of 0.36 m/s (± 0.26 m/s SD) (Table 1). The overall mean drifter bearing was 137.56° (± 44.00° SD) with a mean speed of 0.29 m/s (± 0.19 m/s SD) (Fig. 2B).

Over-ground swimming speed for the subsample of turtles (n = 31) with drifters deployed during their trials was 0.41 m/s (± 0.15 m/s SD) and mean bearing 106.83° (± 21.00° SD) (Fig. S5). The overall mean in-water swimming speed of hatchlings was 0.25 m/s (± 0.09 m/s SD) with a mean bearing of current-corrected tracks of 66.97° (± 31.18° SD) (Fig. 2C). The mean current speed was 0.114 m/s (± 0.0370 m/s SD) during the study’s low flow period, 0.276 m/s (± 0.0405 m/s SD) during the medium flow period, and 0.520 m/s (± 0.111 m/s SD) during the high flow period (Table 2). Percentages of in-water swimming speed to over-ground swimming speed were 80.4% and 26.9% in the *u* and *v* components, respectively, for the highest and lowest flow period days. The in-water hatchling *u* speed was more than twice that during the medium and highest flow periods and was much greater than the in-water *v* speed except during the medium flow period (Table 2). From the linear regression, there was a positive, increasing relationship between over-ground and in-water swimming speeds (*F* = 9.12, df = 1, 29, p = 0.005, adjusted R^2^ = 0.21; Fig. S7).

**Table 2:** Over-ground and in-water hatchling speeds under varying ocean current conditions.

	Low	Medium	High
	8/23/2016	9/03/2016	8/25/2016
East–west (*u*) Over-ground	0.312	0.318	0.439
North–south (*v*) Over-ground	− 0.00954	− 0.0474	− 0.371
East–west (*u*) In-water	0.251	0.108	0.118
North–south (*v*) In-water	0.0581	0.128	0.0147
In-water *u* and over-ground *u* difference	− 0.0601	− 0.210	− 0.321
In-water *v* and over-ground *v* difference	0.0676	0.176	0.386
Mean distance (m) Hatchlings	1631.48	1721.07	2750.62
Mean distance (m) Drifter	611.53	1555.34	2403.78
Mean daily speed of drifter (m/s)	0.114	0.276	0.520

Mean values of east–west (*u*) and north–south (*v*) components of both over-ground and in-water hatchling speed (m/s) in low, medium, and high surface current flows during the study period. Daily mean distance (m) travelled per day by hatchlings with drifters associated with their tracks and drifters are provided, as well as mean daily speed of drifters (m/s).

## Discussion

Estimates of leatherback hatchling speed are rare, making it difficult to calculate the active component of turtle swimming for biophysical models. This study allowed us to assess the effectiveness of the mobile acoustic tracking technique on hatchling leatherback turtles^25^ and provided speed estimates to serve as a foundation for leatherback hatchling dispersal models^26^. The acoustic tracking completion rate was 98%, with 42 hatchling leatherbacks tracked for a mean duration of 83.15 min, showing this methodology can be successfully utilized on leatherbacks in offshore waters. Our mean estimated in-water speed of leatherbacks was the same as the previous estimate of 0.91 km/h (0.25 m/s), while our measured over-ground swimming speed was higher^27^. Our observed mean over-ground swimming speed was 0.41 m/s. The greater speed of the currents relative to the hatchlings’ in-water speed led to their overall movements being strongly influenced by the currents. The ratio of mean animal speed (in-water speed) to flow speed (0.25 m/s/0.29 m/s = 0.86) is within the range described in Chapman et al.^28^ for hatchling sea turtles and indicates how their range of direction relative to the current flow may be restricted.

Hatchlings in lower currents, determined by drifter distance and speed, generally had higher in-water swimming speeds, closer to that of the currents (Table 2). This suggests they were able to partially compensate for the advection caused by the currents, similar to flatback hatchlings in Wilson et al.^23^ and adult leatherback movements as in Shillinger et al.^29^. Hatchlings in higher currents had the greatest difference between over-ground and in-water speed components, suggesting that hatchlings made less of an attempt to resist, or were unable to overcome, these currents.

The stronger the daily nearshore currents, as indicated by the distance and speed the drifters travelled, the greater the surface water influenced hatchling movement, such as during tracking on 25 August 2016 and 2 September 2016 (high surface current flow; 0.520 m/s and 0.474 m/s, respectively) (Table 2). The hatchlings generally moved the farthest when the nearshore current was strong, but they moved in a more southerly direction due to its influence. When the current was weaker, turtles moved more easterly and thus, farther offshore, and travelled a greater distance compared to the drifters. Tracking studies of leatherbacks have shown even adults are influenced by surface currents. The impact of currents will depend on both the animal’s swimming ability and their motivation, whereby foraging opportunities may be presented by current-associated oceanographic features (e.g.^29,30^). Actively swimming hatchlings were shown here to be increasingly influenced by current strength, with resulting movements determined by current flow. During our nearshore tracking, we did not observe any foraging behaviour; hatchlings are expected to feed once they are farther offshore. Movements of hatchlings within currents, therefore, mainly depended on their swimming ability.

The active movement of the hatchlings can play an important role in their dispersal patterns. Over large distances, small changes in directionality influenced by current strength could have large influences on the ultimate destination of the hatchlings^31^. Tidal states can influence currents in many oceanic areas, which have been shown to alter hatchling bearing^23^. This area is not a tidal-dominated system, experiencing relatively small fluxes between multiple daily high and low tidal states (range: − 0.06 to 0.4 m). Over-ground speed of hatchlings was not significantly influenced by tidal state, but a high tidal state and tracking time period (≥ 45 min; Table S2) were weakly associated with increased hatchling bearing, suggesting hatchlings may move more southerly during high tides as they move away from shore. However, given the low variation explained by our model, more data, particularly during high tide, are necessary to better elucidate tidal influence on hatchling directionality near Pacuare. In Putman et al.’s^31^ models, release of young turtles a day apart could have major impacts on the environment encountered and dispersal of individuals. Hatchlings emerging during different tidal states on different days could result in different dispersal outcomes (see Fig. S5).

Vertical movements to a more advantageous rate of flow is a potential adaptive response to currents^28^, a behaviour possibly exhibited by these hatchlings. Some dives performed may have been evasive manoeuvres to avoid airborne predation, although this was not directly quantified, and there were no successful airborne predation attempts during the study. The attachment method used in this study was based on testing to minimise the effect on hatchling movements described in Hoover et al.^25^. Hatchling dive depth at sea was likely constrained to some degree by the line-float-transmitter attachment, which could increase the energy expenditure of this behaviour. During tracking, many hatchlings exhibited diving behaviour and were able to dive deeper than 1.5 m, thus pulling floats underwater. Some turtles dove well below 2 m throughout the trial, and a longer attachment would be recommended to allow full dives to occur. However, while young leatherbacks have the capability of diving deeply^32^, a longer attachment would be a trade-off in increasing drag and difficulty in personnel handling excess line compared to the advantage of decreased inhibition on diving and forward underwater movement.

Given younger leatherback hatchlings have been observed making few U-dives compared to larger, older conspecifics that are foraging for prey, it is unlikely this short tether greatly reduced their forward progress during dives^32^. Further, optimal swimming depth of sea turtles relative to energy expenditure is predicted to be approximately three times a hatchlings’ body length^33,34^, which would constitute a depth of approximately 0.2 m, a near-surface swim. Unless diving deeper to reach an optimal flow zone or avoid predation, hatchlings would ideally swim at this depth. Some hatchlings were observed steadily swimming at or near the surface with a persistent heading, consistent with previous observations of hatchlings^32^. Therefore, we observed anticipated individual variability, where individuals exhibit different swimming strategies regardless of the presence of the line-float-transmitter attachment. Since a direct surface swim would not be energetically efficient, but a near-surface swim is predicted optimal^33,34^, a study examining dive depth would better elucidate the rationale of this behaviour and better inform active dispersal models. Further research on *in-situ* hatchling dive behaviour using new technologies (e.g. video cameras, underwater drones) could provide important insights into the role of currents and other physical processes encountered near the ocean surface throughout their dispersal, an important component to accurate models^31^.

Another adaptation to current flows is for animals to orient within a current^28^. Hatchling compass headings recorded during the trials suggested nearly all turtles targeted a north-easterly offshore retreat from the nesting beach. Visual observations indicated that when hatchlings deviated from this north-eastward trajectory after surfacing from a dive, they redirected themselves. In combination with persistent headings observed by surface-swimming hatchlings, hatchlings seemed to be using mechanical cues of oncoming surface waves or swells for orientation^18,35^. Current-corrected tracks suggest hatchlings headed in an east-northeasterly direction. With the observed north-easterly orientation, hatchlings were likely compensating to overcome the south-southeasterly currents to accomplish easterly, offshore movement.

Remotely sensed fine-scale, nearshore current data is difficult to obtain, with long-term satellite-derived current data estimated at a third of a degree (approximately 37 km) grid resolution^36^. Therefore, we utilised drifters during this experiment to account for the influence of currents on hatchling movement. The drifters generally moved south-southeasterly, suggesting a dominant along-shore current. Further work would benefit from more detailed measurements of coastal currents, for example, using an acoustic Doppler current profiler to provide current flow data within the precise area and depth of individual hatchlings. Our drifters may not have fully captured the flow rate at the average swimming depth of these hatchlings; quantified research on diving behaviour of this population would be warranted to better estimate flow rates encountered. Surface-swimming hatchlings would be more impacted by wind and waves, whereas hatchlings deeper in the water column would be closer to the current flow our drifters measured. While the mobile phone drifter did not have a temperature sensor or live GPS feed, additional components could be easily and inexpensively added. The overall cost was very low (approximately $120 USD), and we did not observe any difference in movement between the two drifters.

The line-float-transmitter attachment always remained behind swimming hatchlings. Tracking required both a combination of visual and acoustic detection, as it was difficult to pinpoint the exact location solely using the directional hydrophone given the wide swath created by a 200 m detection radius and reflections from the boat hull. Periodically, spotting of a lost turtle would occur via the head surfacing, but in most instances, visual recovery relied on the trailing painted floats. The floats were particularly necessary on days with higher sea states (Beaufort sea state 4 or higher) when it was difficult to maintain the boat position relative to hatchling movement, although the low platform height in the small boat also made visual tracking challenging. Overall, the tracking method performed effectively for leatherback hatchlings, and line-float-transmitter attachment removal was easy, immediate, and non-damaging.

The small size and lack of defences of hatchling sea turtles increase predator vulnerability, producing low survival likelihoods^37^, particularly in shallow waters. We were able to witness near attacks that did not ultimately result in predation. The floats appeared to deter predators, such as frigate birds and tarpon, as they veered from the attack on many occasions. In Limón Province, sport fishing is frequent; tarpon, the target of fishers, and seabirds that follow these fishing boats for scraps, may be accustomed to avoiding fishing line and bobbers. Observations of hatchlings avoiding nearby fishes suggests they were still able to overcome possible predation with their attachment. Whether predation was prevented due to these evasive behaviours or the deterrent of the line-float-transmitter attachment is unclear, but the tracking method did not appear to increase the predation risk or mortality, further indicating the efficacy of this attachment methodology for hatchling leatherbacks near Pacuare, Costa Rica.

Short-term tracking techniques provide estimates of directionality, speed, and survival rates of young turtles from a Costa Rican nesting beach that serve as a foundation for dispersal models for this and other populations. The study shows the utility of acoustic tracking of hatchling leatherbacks for fine-scale movement data, which can be incorporated into a biophysical model to understand early dispersal movements, behaviour, and survival of leatherback hatchlings for the Northwest Atlantic population. Comprehension of dispersal components, both active and passive, of young sea turtles extends our understanding of all sea turtle life stages, from developmental habitats to adult foraging ground selection^9,21^. Neritic swimming speeds can provide an estimate of where these hatchlings will be relative to the nesting beach when the dispersal swimming frenzy and yolk reserves run out, an important aspect to understand with transforming ocean conditions under a changing climate and in the context of ever-increasing anthropogenic impacts to marine ecosystems. Leatherback turtles are facing unprecedented population declines, and this information can be used to build knowledge and strengthen conservation efforts vital to preventing extirpation and, ultimately, extinction of this species.

## Methods

### Ethics statement

All procedures for fieldwork in Pacuare Nature Reserve followed approved protocol under Monash University’s School of Biological Sciences Animal Ethics Committee (Protocol No. BSCI/2016/13), the University of Maryland Center for Environmental Sciences’ Institutional Animal Care and Use Committee (IACUC) (Research Protocol No. S-CBL-16–11), and the Costa Rican Ministerio Del Ambiente y Energia, Sistema Nacional de Áreas de Conservación (SINAC), Área de Conservación La Amistad Caribe (ACLAC) (RESOLUCIÓN SINAC-ACLAC-PIME-VS-R-022-2016; RESOLUCIÓN SINAC-ACLAC-PIME-VS-R-025-2016). The study was performed in accordance with the approved guidelines.

### Hatchling tracking

To examine in-situ factors of turtle dispersal into the offshore environment, leatherback hatchlings were tagged with coded acoustic transmitters between 20 August and 3 September 2016 in Pacuare Nature Reserve, Limón Province, Costa Rica (Fig. 1). At Pacuare, hatchlings were obtained from hatchery, incubator-reared, and relocated nests. Hatchery nests consisted of eggs collected as they were laid, transported in plastic bags for less than 5 km, and reburied in two separate protected areas. In these protected, monitored enclosures, the eggs were safeguarded (e.g. from predation) and otherwise developed naturally. Relocated eggs were collected from nests laid the night prior, transported in plastic bags a short distance above the high tide line, reburied on the nesting beach and unmonitored thereafter, until such time as hatchlings emerged. Hatchlings from hatchery and relocated nests were collected as they naturally emerged from the buried nests. Incubator-reared eggs were collected as they were laid, transported up to 1 km in vacuum-sealed bags, and raised under 3 treatments: control, low-oxygen, and high-oxygen in accordance with the Williamson^38^ protocol. Eggs were incubated for the first 5 days of development in: hypoxia (1% O^2^) for the low-oxygen treatment and hyperoxia (42% O^2^) for the high-oxygen treatment. As they did not have to expend time and energy exiting a nest, incubator-hatched turtles were left to absorb their yolk for 2 days^38^. Turtles held post-emergence from their nest (hatchery and relocated hatchlings) or eggs (incubator-reared hatchlings) were kept in moistened, sand-lined incubators at approximately 30 °C to reduce energy expenditure prior to trial release and prevent potential decreases in swimming performance^39^. To minimise the influence of genetic relatedness, hatchlings were taken from all available nests (n = 9 in total from hatchery, relocated, and incubator nests) at the time of the study, resulting in parentage by nine females. Turtles were weighed and measured prior to trials using a scale and calliper. To prevent overheating on the boat, turtles were transported in a bucket covered by a wet towel with a moistened cloth inside.

Acoustic tracking was conducted using Vemco V5-180 kHz transmitter tags (0.38 g in-water weight; 0.65 g in-air weight) and tethered to the turtle via a line-float-transmitter assembly (6.85 g in-air weight) and Vetbond based on Gearheart et al.^24^ and Hoover et al.^25^ (Fig. S1). The monofilament line in the line-float-transmitter assembly was a total length of 2 m; the first float was suspended 1.5 m behind the hatchling, and the second float was an additional 0.5 m. The brightly coloured orange floats (4.4 cm by 1.9 cm) allowed for visual tracking in the water when acoustic signal was insufficient. Tracking began outside the surf zone, approximately 0.4 km from shore, where turtles were taken via a small 6 m, 150 hp motorboat. The release location was the approximate midpoint of the two hatcheries where hatchlings were collected. Between sunrise and sunset, each turtle was followed at a distance of 10–20 m in the boat using a Vemco VR100 acoustic receiver and VH180-D-10M directional hydrophone^22^. The V5 tag detection range was approximately 200 m. The VR100 receiver stored the detections, and the data were downloaded to reconstruct hatchling movement paths. The mobile acoustic receiver allowed tracking of the turtles’ movements for a longer period and over a broader area than visual tracking alone because turtles were found acoustically when visual contact was lost.

Hatchlings were tracked only during daylight hours over a 3-week period given hatchling and boat availability. Although hatchlings generally emerge during cooler, evening hours of the day in Costa Rica, no effect on the overall innate behaviour of hatchlings was anticipated^18,40^. The tracking data should still be indicative of the orientation and speed at which hatchlings are likely to swim. Nighttime tracking was logistically infeasible because of the hazards associated with the oceanic entry point. For a track to provide enough data for inclusion in the analysis, a minimum tracking time of 30 min was established. Turtles were tracked individually for approximately 90 min. Track duration was a trade-off between obtaining a large sample of tracks to account for individual variability, while providing robust speed and orientation information. At the end of each track, the turtle was recovered with a small net, the line-float-transmitter attachment was completely removed, and the turtle was released at the recovery location. The Velcro piece easily removed from the carapace, and there were no evident damages, marks, or lesions from this attachment method on the leatherback hatchlings. Handling was kept to a minimum to reduce any unnecessary stress on the turtles.

### Surface current trajectories

Two drifters were used to obtain data on local sea surface currents to evaluate the effect of currents on hatchling movements. A Pacific Gyre Microstar drifter was deployed at the beginning of each turtle track (Fig. S2A). The drifter’s surface float was equipped with a GPS unit that used the iridium short burst data service to broadcast location coordinates every 5 min. A flag was attached to the surface float for increased visibility. Sea surface temperature was recorded by the drifter with a Pacific Gyre probe with 0.1 °C accuracy. The position and temperature data of each drifter release were retrieved from the Pacific Gyre website (https://www.pacificgyre.com). One drifter track was removed from analysis because it entered the surf zone and did not represent nearshore surface currents.

A secondary drifter was launched when equipment permitted at the approximate halfway point during tracking of a turtle. This better estimated the immediate currents the hatchling was experiencing and was used to estimate shifts in the nearshore currents as the turtles headed offshore. This second drifter was constructed using a Davis Instruments aluminium radar reflector with 80 cm of parachute cord attached to a 20.3 cm diameter Panther Plast trawl float (Fig. S2B). The centre of the drifter sat 1 m from the water’s surface, similar to the depth of the Microstar drifter. A piece of wood affixed to the top of the float had a Samsung Galaxy Core Prime mobile phone attached in a waterproof bag. A GPS application was started with each drifter release to provide locations at one minute intervals. Foam tubing was zip-tied around the middle of the trawl float to maintain the GPS unit in an upright position. The float had a flag attached for visibility on the water. Positions were stored on the mobile phone and downloaded upon retrieval of the drifter. Both drifters were recovered at the completion of each individual turtle track.

### Environmental data

To understand the influence of tidal states and bathymetry on local currents experienced by hatchling leatherbacks, daily tidal currents were obtained at Limón, Costa Rica (10.00° N, 83.03° W; https://tides.mobilegeographics.com). Periods of peak tides (i.e. high and low) were defined as one hour before and after the measured minima or maxima, with ebb and flow tides between those periods of peak tides. Tidal states were categorised as: high, ebb, low, and flow (Fig. S3A). High-resolution, near-shore bathymetry data were obtained at a 0.0011° resolution from the Global Multi-Resolution Topography Synthesis dataset (https://www.gmrt.org). Missing values were filled with data from the General Bathymetric Chart of the Oceans dataset (GEBCO-2014; https://www.gebco.net; 0.0042° resolution). Bathymetric values for each hatchling track were extracted with a ‘bilinear’ interpolation in the R ‘raster’ package^41^ (Fig. S3B). All analyses were conducted in the R environment^42^.

### Hatchling movement analysis

An acclimation period of five min was applied to each turtle track to provide time for the hatchling to orient and adjust to the water temperature. Intervals greater than five min between recorded hatchling positions were removed to prevent erroneous calculations (0.03% of recorded positions). These time lapses occurred when the boat was actively searching for a hatchling. Despite the combination of surface floats and the directional hydrophone, maintaining visual and acoustic contact with turtles was challenging, even in calm waters. Many hatchlings dove for short periods (< 60 s); when contact with a hatchling was lost, the visual observer would maintain watch on the last known bearing. The boat would ‘dead reckon’ the hatchling position, maintaining speed and heading until visual contact was re-established. Distances resulting in over-ground speeds greater than 0.75 m/s (0.02% of positions) were removed as spurious positions because they were extreme outliers and inconsistent with adjacent and published values^8,27,43^. Final positions were mapped using the ‘mapdata’ package in the R statistical software (R Version 3.5.2; https://www.R-project.org)^44^.

To correct for boat movement as it repositioned relative to the hatchling to maintain a 10–20 m distance, mean latitude and longitude values were calculated for every five-minute period. This provided a regularised track representative of hatchling movement throughout the study period from which distance and speed were calculated. Drifter distances were calculated using the GPS locations from the Microstar surface float GPS and the mobile phone GPS at five min intervals. After converting these distances to speed, values exceeding 1.0 m/s (n = 7) were removed as they were inconsistent with adjacent values, mainly occurring at the beginning and end of tracks. The ‘argosfilter’ package in the R statistical software was used in distance and bearing calculations^45^. Mean bearing and heading were calculated with circular statistics.

Over-ground speed of hatchlings was calculated based on the total distance travelled during every 5-min interval of each hatchling track, represented as observed distance (m) over time (s). This over-ground speed is the apparent speed of the turtle moving through the water, which includes the turtle’s movements and that of the surface water. The speed of the drifter was calculated in the same manner.

Variations in the over-ground swimming speed of hatchlings were examined using a linear mixed effects model framework. The response variable of over-ground speed was square-root transformed based on results of a Box-Cox transformation to meet model assumptions. The explanatory variables of the model were categorical time as five-minute intervals (Time 1 = 0–5 min, Time 2 = 5–10 min, etc.), treatment (control, low-oxygen, high-oxygen, and combined hatchery-reared and relocated nests), bathymetry, and tidal state, and interactions between these variables were tested. The time variable was restricted to the first 85 min of each track because only a few turtles were tracked for longer durations. Hatchery-reared and relocated nests were treated as one treatment due to the low sample size of relocated nests; hatchlings from those nests experienced a natural emergence onto the beach under similar rearing conditions, with no anticipated differences. Parentage and day of release were confounded with treatment and were not examined. The best fit model was selected based on the model with the lowest Akaike information criterion. Fitting the model runs with restricted maximum likelihood, the chosen random effects structure was intercepts varying amongst individuals, and the error structure was an autoregressive lag 2, suggesting a dependency 2 time lags apart. ANOVA F-tests were used to test for significance of model effects, and ANOVA summary statistics were calculated to describe effects.

Changes in the bearing of hatchlings were examined in the same manner as over-ground speed, with bearing as the response variable. Diagnostic checks were performed on model residuals, which indicated a linear mixed effects model was suitable after data transformation. Squared bearing was run as a result of the Box-Cox transformation with explanatory variables of categorical time, treatment, bathymetry, and tidal state. The best fit linear mixed effects model included random effects differing by individual and an autoregressive lag 2 error structure.

### Effects of surface currents on swimming speed and bearing

To obtain a value for the true swimming speed component of hatchling leatherbacks, the surface water flow in which the turtles were swimming was removed from the measured speed of the turtle^46^. This in-water swimming speed was calculated as the difference of the hatchling over-ground velocity and the velocity of the surface currents, as estimated by the drifters in this study. Over-ground speed of hatchlings and drifters was broken into velocity components using equations in Bailey et al.^47^, which accounted for each turtle’s speed and bearing to obtain east–west (*u*) and north–south (*v*) components. The nearest five-minute intervals of each hatchling were matched with the corresponding drifter released. Some hatchlings (n = 11) did not have drifters deployed with them due to equipment issues and were not included in this portion of the analysis. For turtles with two drifters launched during the trial, one in the beginning and one in the middle, the second drifter’s data were used once recording started. Where a single drifter was deployed, those data were used for analysis throughout the track. This provided surface current values closer to those directly experienced by the hatchlings at each given time interval. The drifter’s *u* and *v*-velocity components were differenced from each hatchling’s corresponding over-ground speed components. The in-water speed of the hatchlings was then defined as the square root of the sum of the squared *u* and *v*-velocities. To determine true bearing for each location, in-water speed components were then used to correct hatchling tracks for currents from equations in Gaspar et al.^46,48,49^.

Mean current speeds were used to compare hatchling movements from tracks during the lowest, middle, and highest flow periods from the study. The relationship between over-ground and in-water swimming speed was examined using a linear regression on the mean values from each hatchling.

## Supplementary information

Supplementary Information.
