# Supplementary material for: Nearshore neonate dispersal of Atlantic leatherback turtles (Dermochelys coriacea) from a non-recovering subpopulation
Source: Sci Rep. 2020 Oct 30;10:18748. doi: 10.1038/s41598-020-75769-0 (PMC7603482; doi:10.1038/s41598-020-75769-0)
Supplement: Supplementary file 1 — Supplementary Information. [file 41598_2020_75769_MOESM1_ESM.docx]

# Nearshore neonate dispersal of Atlantic leatherback turtles (*Dermochelys coriacea*) from a non-recovering subpopulation

**Aimee L. Hoover, George L. Shillinger, Sean A. Williamson, Richard D. Reina, and Helen Bailey**

Supplementary Information


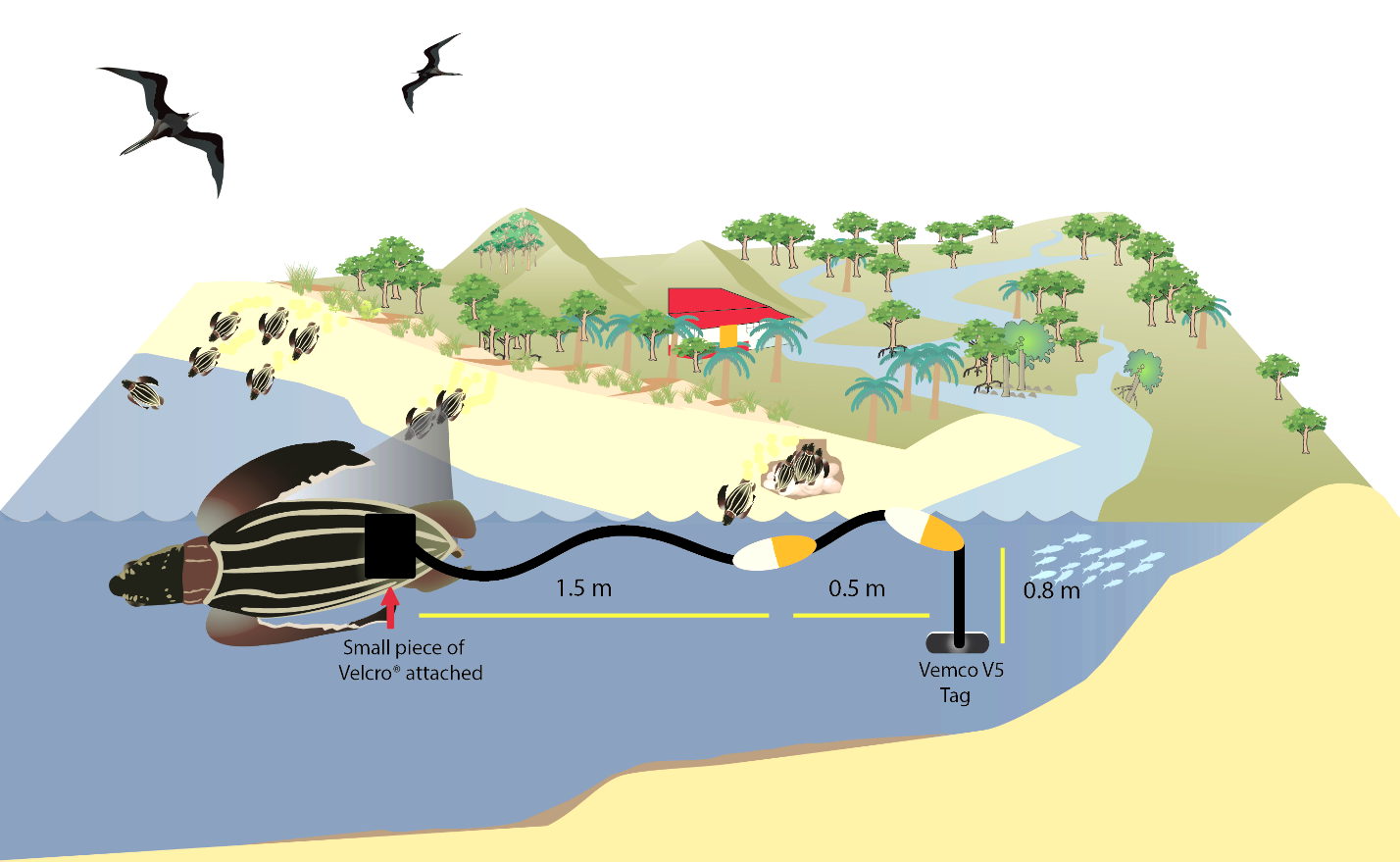


**Fig. S1. Acoustic line-float-transmitter attachment method used on leatherback hatchlings.** Symbols courtesy of the Integration and Application Network, University of Maryland Center for Environmental Science (ian.umces.edu/symbols/).


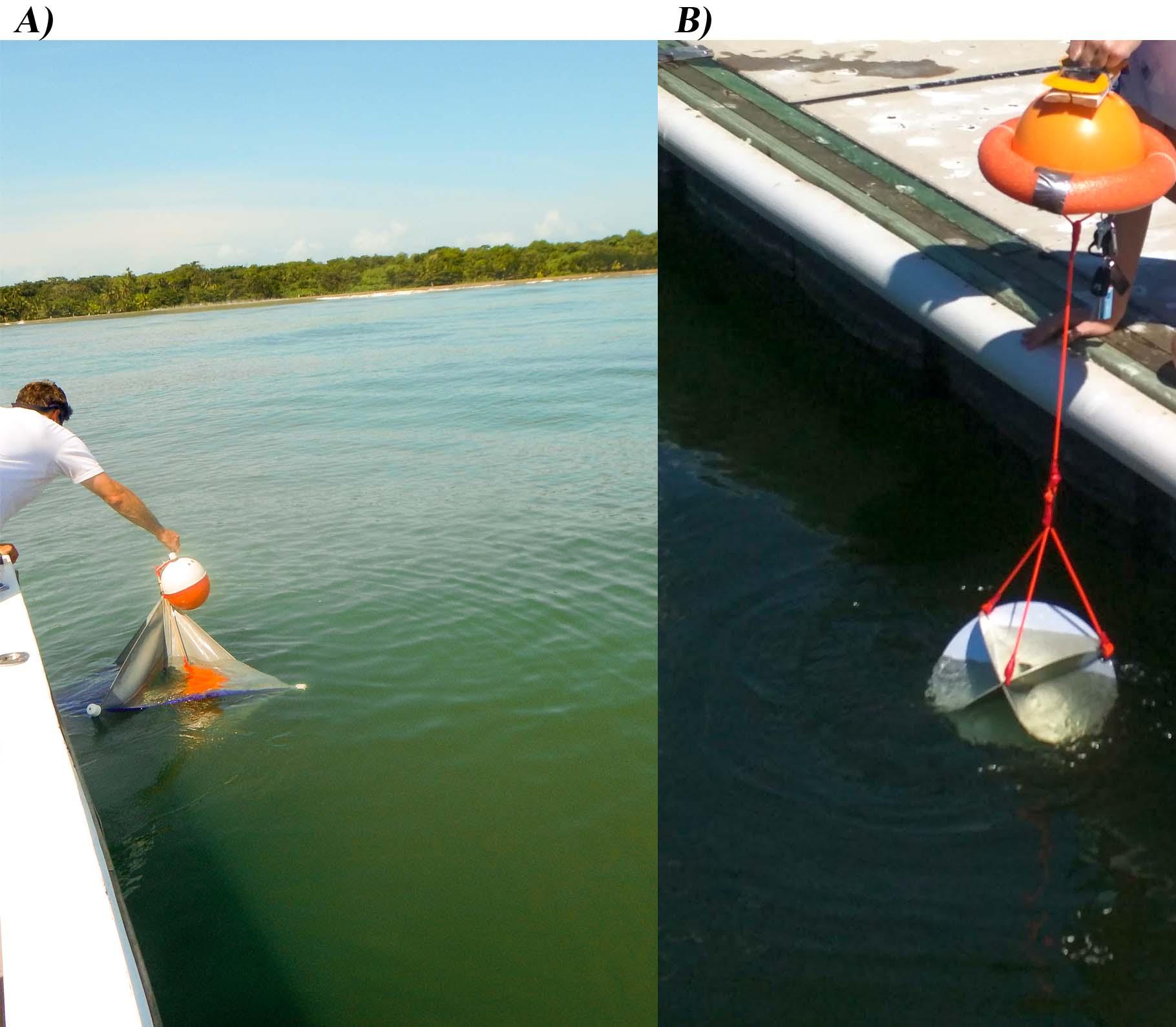


**Figure S2**. **The drifters launched during the hatchling tracking to measure surface currents.** (**A**) The Pacific Gyre Microstar drifter launched nearshore of Pacuare Nature Reserve, Costa Rica prior to starting a hatchling track. (**B**) The surface drifter design with a mobile phone to record GPS locations launched at the midpoint of hatchling tracks.

**
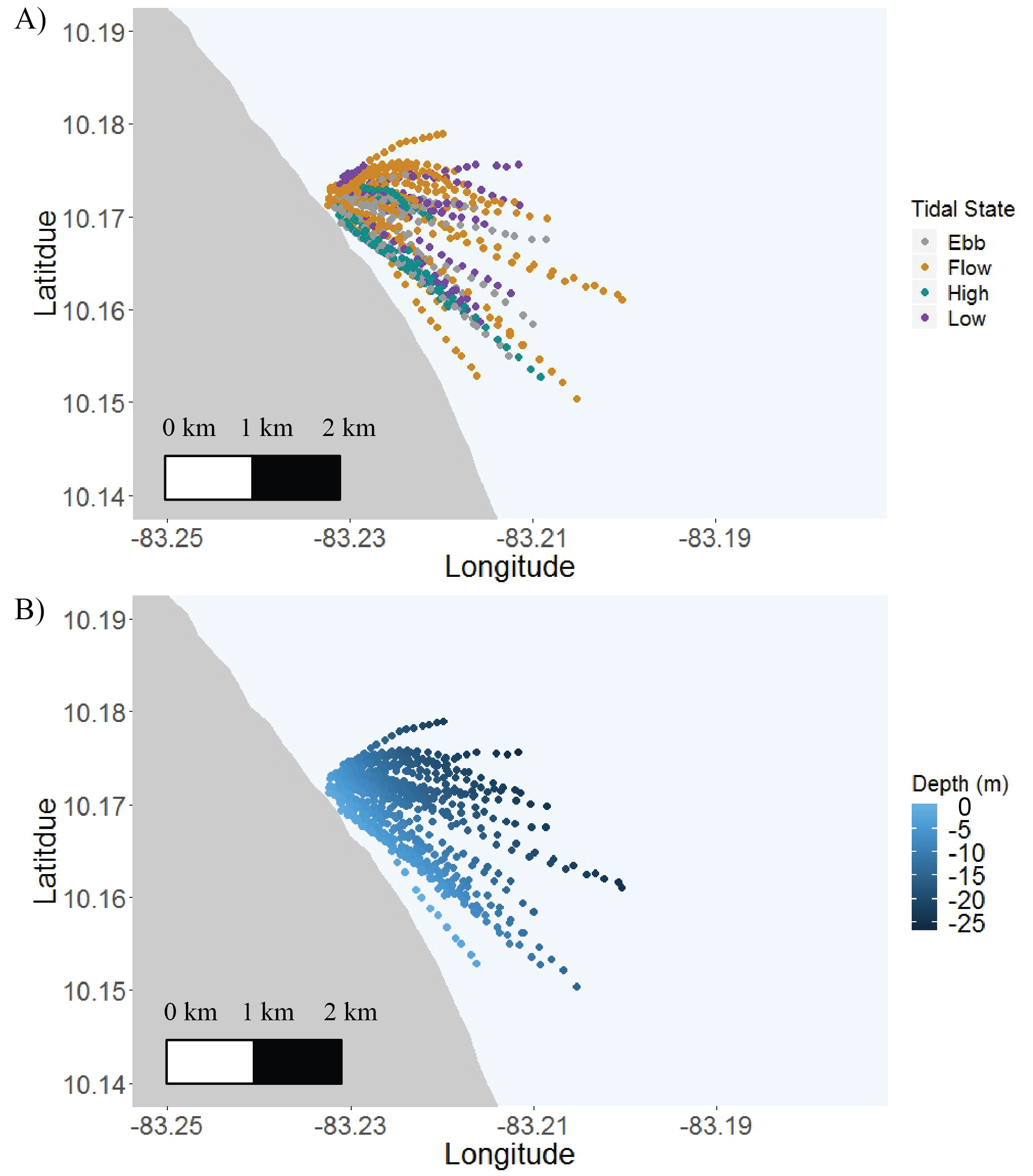
Figure S3**. **Maps of the environmental variables.** Values for (**A**) tidal state and (**B**) bathymetry (m) at each mean five-minute hatchling observation.

**
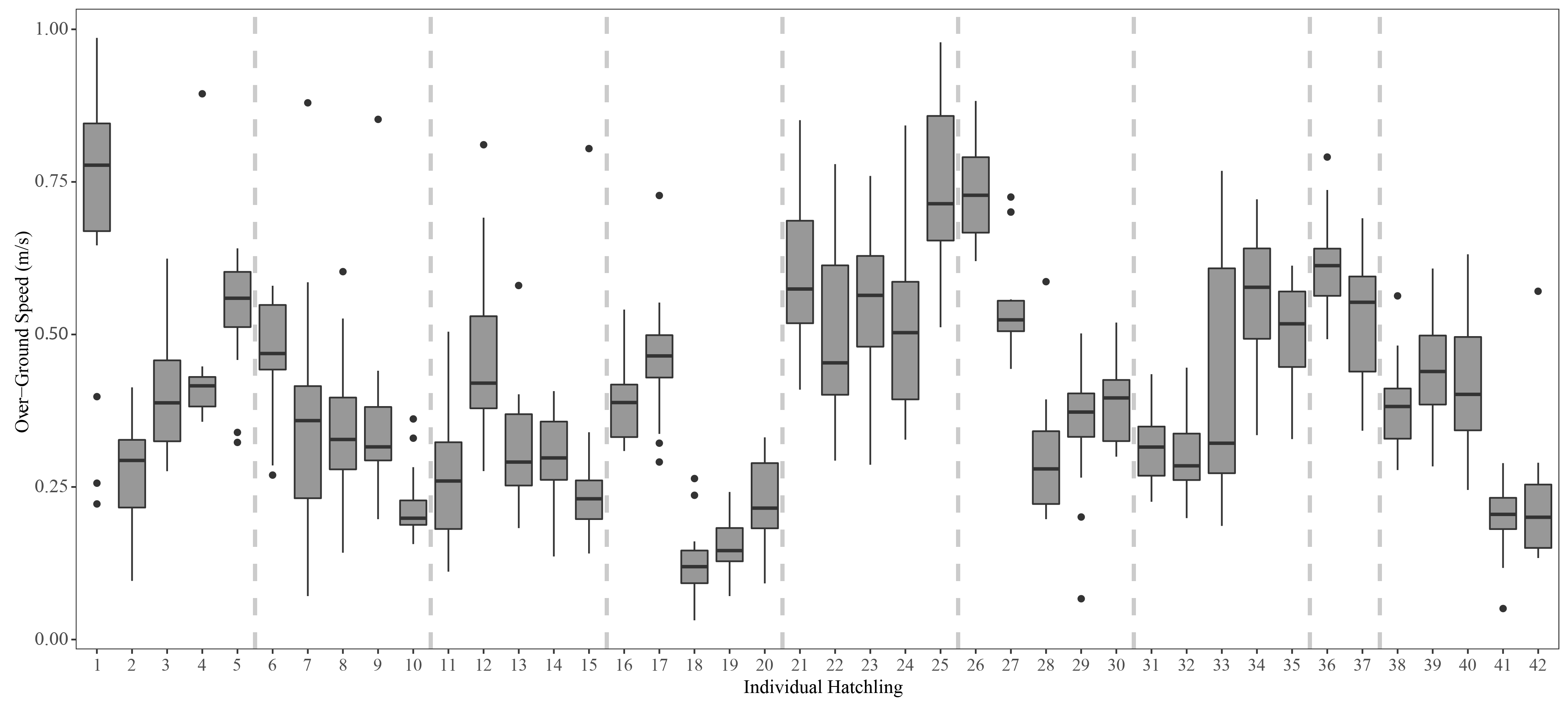
**

**Figure S4**. **Box-and-whisker plot of leatherback hatchlings’ over-ground speed (m/s).** Gray vertical lines indicate different release dates.


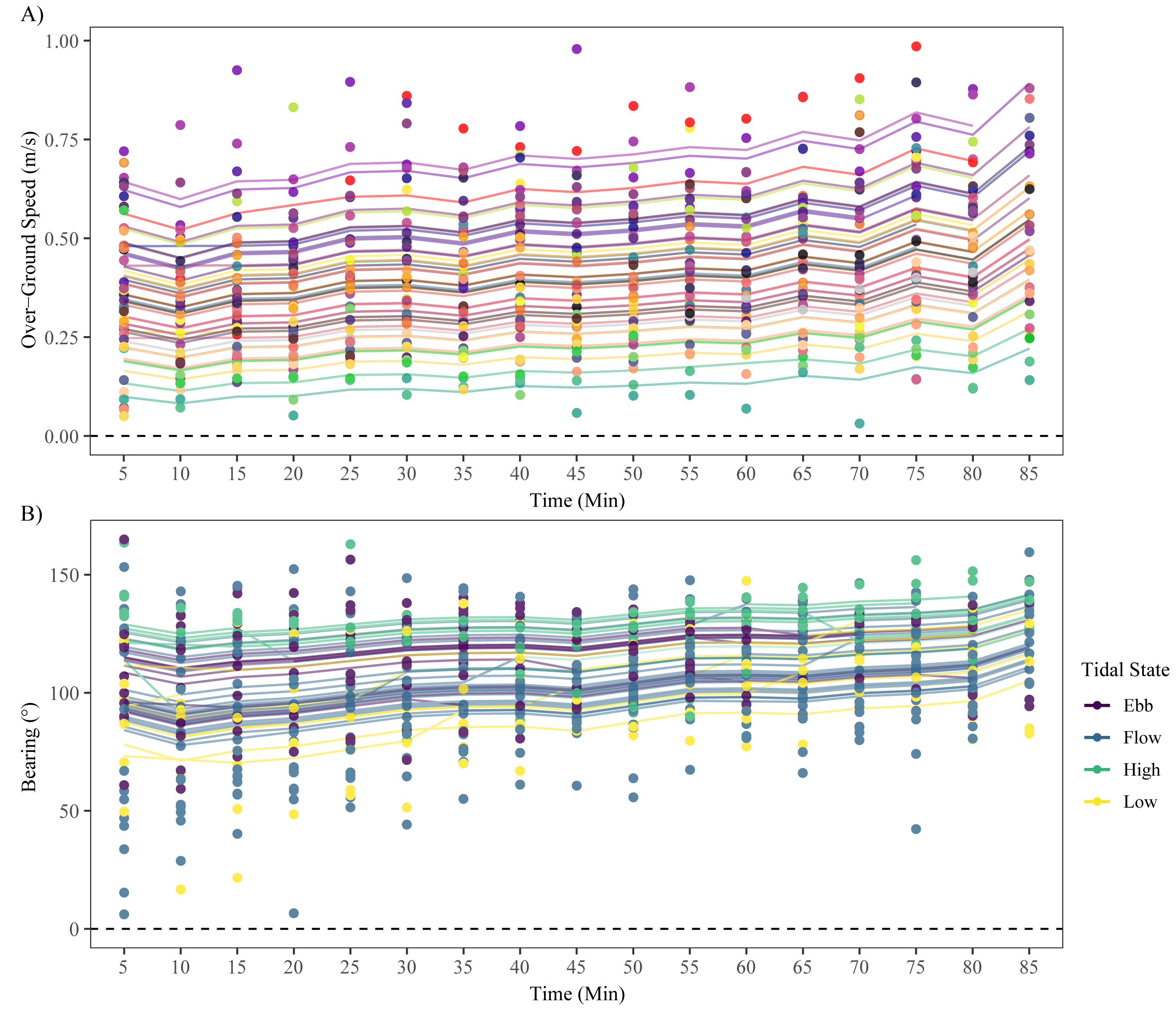


**Figure S5**. **Linear mixed effects model results for the hatchling over-ground speed and bearing across time.** Hatchling (**A**) over-ground speed (m/s), with colour differing for each individual, and (**B**) bearing (degrees) by tidal state. Lines indicate predicted model fit.


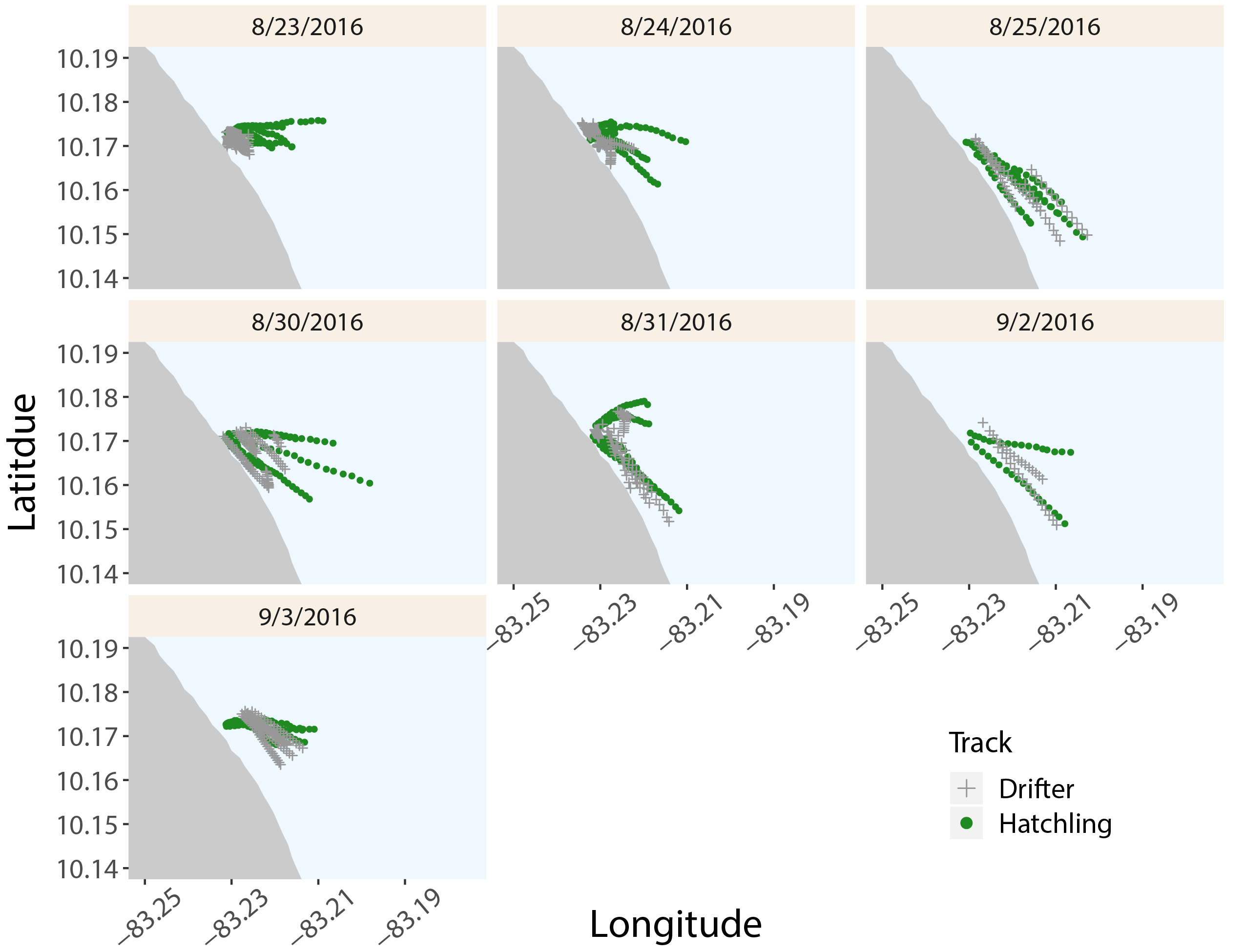


**Figure S6**. **Tracks of drifters and hatchlings near Pacuare Nature Reserve, Costa Rica.** Tracks of drifters (gray ‘plus’ symbols) and hatchlings (green dot symbols) by date of release. The movement of the drifters represented the nearshore surface drift encountered by the hatchlings.


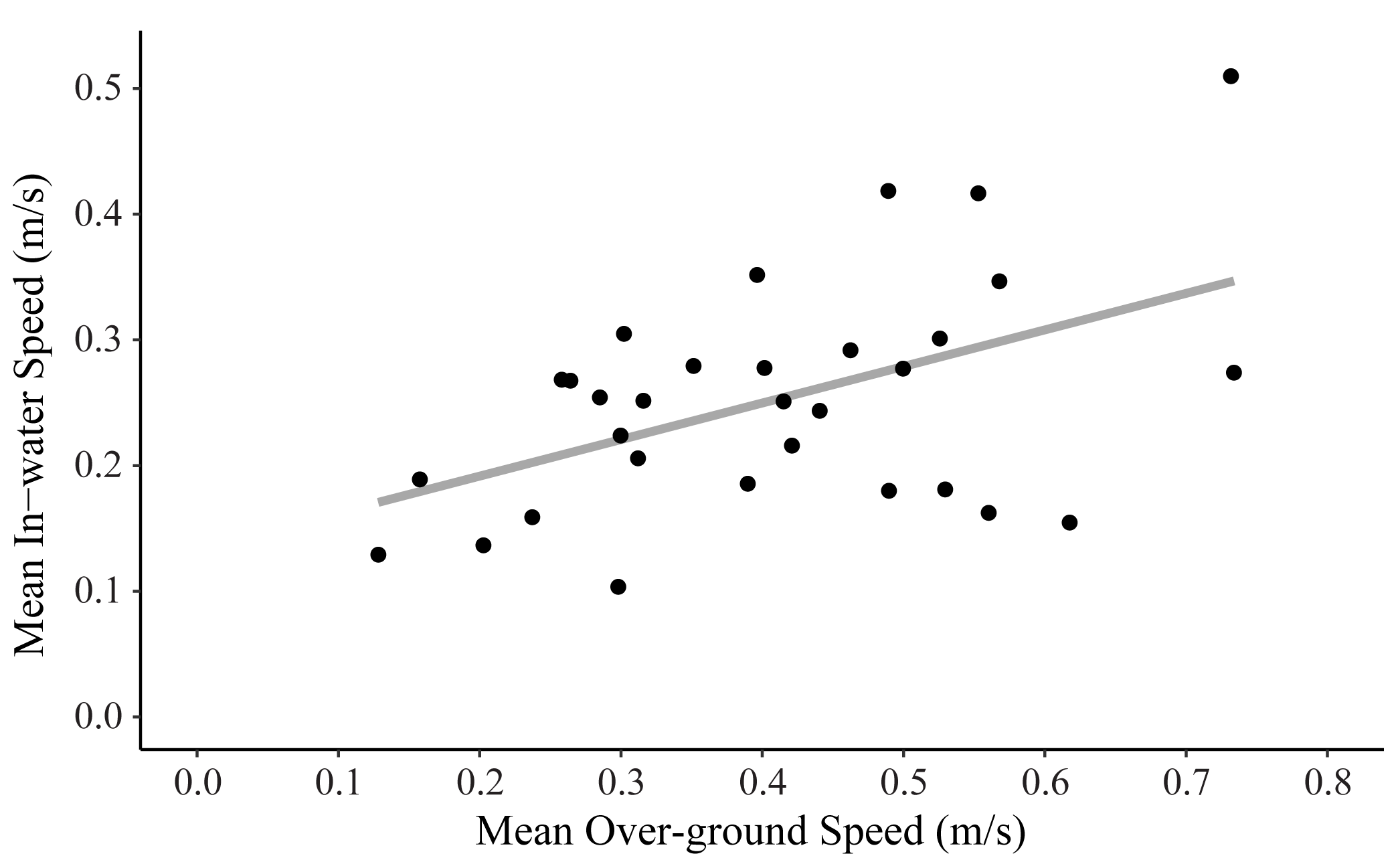


**Figure S7**. **Linear regression plot for hatchling over-ground speed and in-water speed.**

Individual hatchling mean over-ground speed (m/s) plotted against mean in-water speed (m/s), with regression line shown in gray.

**Table S1**. **Generalized linear mixed model results for hatchling over-ground speed in relation to tracking time.** Hatchling over-ground square-root transformed speed (m/s) was modeled as a function of time as five-minute intervals. Each model was fit with an auto-regressive lag 2 correlation structure and random effects amongst individuals. An asterisk denotes statistical significance at p < 0.05.

| **Response** | **Explanatory Factors** | **Degrees of Freedom** | **F-value** | **P-value** |
| --- | --- | --- | --- | --- |
| Square-root of speed (m/s) | Intercept | 1 | 1100.02 | < 0.0001* |
|  | Time | 16 | 6.04 | < 0.0001* |
| Bearing squared (°) | Intercept | 1 | 412.93 | < 0.0001* |
|  | Time | 3 | 7.11 | < 0.0001* |
|  | Tidal State | 16 | 3.08 | < 0.0001* |

**Table S2**. **Results of the mixed effects models for over-ground hatchling speed and bearing.** The hatchling over-ground speed ((m/s)^0.5^) and bearing (degrees^2^) models were fit with an auto-regressive lag 2 correlation structure and random effects amongst individuals. An asterisk denotes statistical significance at p < 0.05. Each five-minute interval amongst the fixed effects is shown in parentheses.

|  | Square-Root of Over-Ground Speed | | | | Squared Bearing | | | |
| --- | --- | --- | --- | --- | --- | --- | --- | --- |
| **Fixed Effects** | Estimate | SE | t-value | p-value | Estimate | SE | t-value | p-value |
| Intercept | 0.580 | 0.022 | 26.10 | 0.00* | 9858.36 | 1006.12 | 9.80 | 0.00* |
| Flow Tide | NA | NA | NA | NA | 1333.28 | 964.41 | 1.38 | 0.17 |
| High Tide | NA | NA | NA | NA | 3671.38 | 1067.40 | 3.44 | < 0.001* |
| Low Tide | NA | NA | NA | NA | -673.25 | 862.99 | -0.78 | 0.44 |
| Time 2 (5 – 10) | -0.028 | 0.017 | -1.66 | 0.098 | -1033.41 | 527.47 | -1.96 | 0.051 |
| Time 3 (10 – 15) | 0.001 | 0.015 | 0.051 | 0.96 | -410.74 | 542.59 | -0.76 | 0.45 |
| Time 4 (15 – 20) | 0.003 | 0.017 | 0.18 | 0.86 | -122.49 | 620.43 | -0.20 | 0.84 |
| Time 5 (20 – 25) | 0.028 | 0.017 | 1.63 | 0.10 | 433.51 | 658.71 | 0.66 | 0.51 |
| Time 6 (25 – 30) | 0.030 | 0.018 | 1.69 | 0.091 | 989.75 | 697.54 | 1.42 | 0.16 |
| Time 7 (30 – 35) | 0.019 | 0.018 | 1.04 | 0.30 | 1202.18 | 727.65 | 1.65 | 0.099 |
| Time 8 (35 – 40) | 0.041 | 0.018 | 2.24 | 0.025* | 1247.78 | 751.47 | 1.66 | 0.097 |
| Time 9 (40 – 45) | 0.035 | 0.018 | 1.94 | 0.053 | 933.14 | 770.99 | 1.21 | 0.23 |
| Time 10 (45 – 50) | 0.042 | 0.018 | 2.30 | 0.022* | 1579.12 | 786.44 | 2.01 | 0.045* |
| Time 11 (50 – 55) | 0.053 | 0.018 | 2.90 | 0.004* | 2238.85 | 799.01 | 2.80 | 0.005* |
| Time 12 (55 – 60) | 0.049 | 0.018 | 2.64 | 0.008* | 2289.83 | 812.67 | 2.81 | 0.005* |
| Time 13 (60 – 65) | 0.075 | 0.018 | 4.10 | 0.00* | 2165.02 | 819.87 | 2.64 | 0.009* |
| Time 14 (65 – 70) | 0.063 | 0.018 | 3.42 | < 0.001* | 2614.87 | 826.04 | 3.17 | 0.002* |
| Time 15 (70 – 75) | 0.100 | 0.018 | 5.57 | 0.00* | 2826.09 | 834.81 | 3.39 | < 0.001* |
| Time 16 (75 – 80) | 0.084 | 0.019 | 4.46 | 0.00* | 3216.03 | 850.35 | 3.78 | < 0.001* |
| Time 17 (80 – 85) | 0.160 | 0.021 | 7.48 | 0.00* | 4941.92 | 913.94 | 5.41 | 0.00* |
